# Evaluation of LMP1 of Epstein-Barr virus as a therapeutic target by its inhibition

**DOI:** 10.1186/1476-4598-9-184

**Published:** 2010-07-09

**Authors:** Adele Hannigan, Joanna B Wilson

**Affiliations:** 1 Division of Molecular and Cellular Biology, Faculty of Biomedical and Life Sciences, University of Glasgow, Glasgow G11 6NU, UK; 2 Current address: EnGeneIC Ltd, Sydney, NSW 2066, Australia

## Abstract

**Background:**

The latent membrane protein-1 (LMP1) encoded by Epstein-Barr virus (EBV) is an oncoprotein which acts by constitutive activation of various signalling pathways, including NF-κB. In so doing it leads to deregulated cell growth intrinsic to the cancer cell as well as having extrinsic affects upon the tumour microenvironment. These properties and that it is a foreign antigen, lead to the proposition that LMP1 may be a good therapeutic target in the treatment of EBV associated disease. LMP1 is expressed in several EBV-associated malignancies, notably in Hodgkin's lymphoma and nasopharyngeal carcinoma (NPC). However, the viral protein is only detected in approximately 30%-50% of NPC samples, as such its role in carcinogenesis and tumour maintenance can be questioned and thus its relevance as a therapeutic target.

**Results:**

In order to explore if LMP1 has a continuous function in established tumours, its activity was inhibited through expression of a dominant negative LMP1 mutant in tumour cell lines derived from transgenic mice. LMP1 is the tumour predisposing oncogene in two different series of transgenic mice which separately give rise to either B-cell lymphomas or carcinomas. Inhibition of LMP1 activity in the carcinoma cell lines lead to a reduction in clonagenicity and clone viability in all of the cell lines tested, even those with low or below detection levels of LMP1. Inhibition of LMP1 activity in the transgenic B-cell lines was incompatible with growth and survival of the cells and no clones expressing the dominant negative LMP1 mutant could be established.

**Conclusions:**

LMP1 continues to provide a tumour cell growth function in cell lines established from LMP1 transgenic mouse tumours, of both B-cell and epithelial cell origin. LMP1 can perform this function, even when expressed at such low levels as to be undetectable, whereby evidence of its expression can only be inferred by its inhibition being detrimental to the growth of the cell. This raises the possibility that LMP1 still performs a pro-oncogenic function in the 50% to 70% of NPC tumours wherein LMP1 protein expression cannot be detected. This reinforces the basis for pursuing LMP1 as a therapeutic target in EBV associated LMP1-expressing malignancies.

## Background

Epstein-Barr Virus (EBV) is a human herpes virus which is associated with a number of malignant diseases reflecting the viral tropism primarily to B-cells but also to epithelial cells and rarely other cell types. The EBV-associated B-cell cancers include endemic Burkitt's lymphoma (BL), a subset of Hodgkin's disease (HD) cases and lymphoid tumours arising in immunosuppressed patients; the epithelial cell cancers include nasopharyngeal carcinoma (NPC) and a proportion of gastric cancers. EBV shows a different but typical pattern of latent gene expression in each of these malignancies, from the most restricted pattern of viral expression in BL, to expression of all of the viral latent genes in post-transplant lymphoproliferative disease. NPC and HD biopsies show an intermediate pattern of viral gene expression involving EBNA-1, latent membrane proteins-1 and -2A (LMP1 and LMP2A), EBERs and the BART micro RNAs [1].

LMP1 exhibits properties of a classical oncoprotein, inducing promotion of cell growth and inhibition of apoptosis in a variety of cell types *in vitro *[2]. In addition it has been demonstrated to contribute to both B-cell and epithelial cell tumourigenesis *in vivo *in transgenic mice [3-5]. LMP1 achieves its wide ranging phenotypic effects through the activation of multiple signalling cascades. It activates the NF-κB, JNK and JAK/STAT pathways through direct interaction with pathway intermediary proteins [6]. As a consequence of the gene expression changes induced, for example affecting EGFR and it's ligands [7,8], further pathways are triggered including the ERK/MEK and p38/MAPK pathways. As such, LMP1 is considered as the primary oncogene of the virus and a likely candidate in driving the development of several of the EBV associated malignancies.

Significant progress has been made in recent years in cancer therapeutics in the design of inhibitory molecules that impact relevant signalling pathways, for example B-Raf inhibition in the treatment of melanoma [9]. As a foreign antigen that constitutively activates multiple pathways, LMP1 represents a good therapeutic target in the treatment of EBV associated malignancies. Moreover, while LMP1 activates growth pathways within the cancer cell, in deregulating NF-κB it also impacts a seminal pathway in inflammation programmes and thus potentially, factors in the tumour microenvironment. Therefore targeting LMP1 could affect both intrinsic and extrinsic factors essential to tumour growth. LMP1 expression has been confirmed by immunohistochemical studies in EBV-associated HD. However, detection of LMP1 protein in NPC biopsies is highly variable, with only between 30% to 50% of tumours showing clear expression [10] despite the detection of LMP1 RNA in most samples. Indeed it has been shown that the BART micro RNAs of the virus, which are abundantly expressed in NPC, negatively regulate LMP1 protein expression [11]. This raises some uncertainty about the role of LMP1 in the genesis of NPC and particularly any tumour maintenance function, especially in those tumours where expression cannot be detected. This in turn poses the question of whether LMP1 is a rational therapeutic target.

Inhibition of LMP1 expression by siRNA in an EBV positive NPC derived cell line C666-1, which clearly expresses LMP1, was found to induce cell cycle arrest and enhance the sensitivity of the cells to cisplatin [12]. This observation is encouraging with respect to LMP1 as a potential therapeutic target. However it is unknown at present if this finding will be limited to those NPC tumours with high LMP1 expression. In this study we sought to evaluate the impact of LMP1 inhibition in multiple cell lines, of both epithelial and B-cell origin where LMP1 was the driving oncogene in the development of the tumour. It is notoriously difficult to derive cell lines from NPC and HD tumours and as a consequence there are few lines available. To this end we used cell lines derived from tumours from transgenic mice where LMP1 was the predisposing oncogene. These lines were also used with a view to future *in vivo *drug testing. In all of the LMP1 transgenic cell lines tested, inhibition of LMP1 activity inhibited the growth properties of the cells (to varying extents in the different lines) surprisingly even in those where LMP1 protein expression was not detectable. Firstly, this demonstrates that even extremely low levels of LMP1 can continue to provide a growth advantage to cancer cells and secondly, as a consequence, its inhibition could be an effective route in the treatment to eliminate the cells. However in one highly malignant carcinoma cell line, inhibition of LMP1 lead to the selection of escape mutants indicating that any treatment targeting LMP1 would be best used as part of a combined therapy regime.

## Results

### LMP1 expression in transgenic carcinoma and lymphoma cell lines

In order to investigate the tumour growth promoting properties of LMP1 and whether its continued expression is required in established tumours, carcinomas and B-cell lymphomas from LMP1 expressing transgenic mice were established in culture.

**Figure 1:**
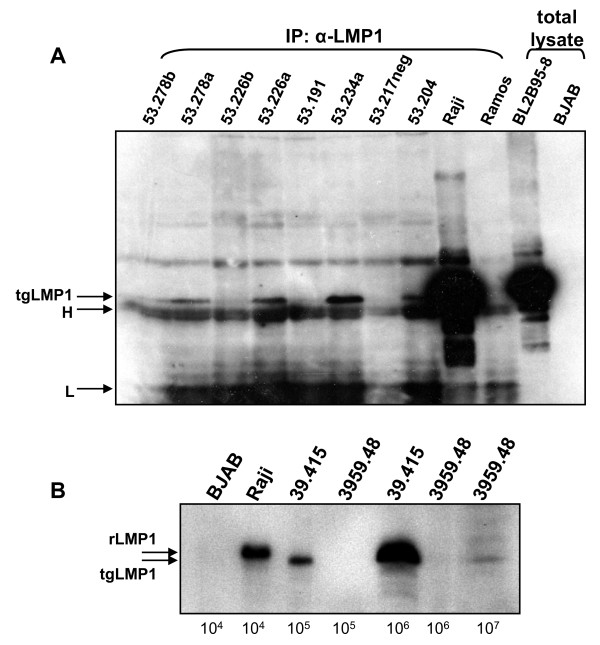
**LMP1 expression in transgenic mouse derived cell lines**. (A) LMP1 was immunoprecipitated from 300 μg of urea buffer-extracted proteins from seven PyLMP1 transgene-positive line 53 carcinoma cell lines and one transgene-negative cell line (as denoted 53.217neg) and 100 μg of protein from control cell lines: Raji (EBV +ve, containing 50-60 copies of EBV) and Ramos (EBV -ve), using the S12 antiserum. Immunoprecipitated proteins were separated by 10%SDS-PAGE and blotted alongside two total lysate (boiling mix extracted) controls, BL2B95-8 (EBV +ve) and BJAB (EBV -ve) (1 × 10^5 ^cells each). The blot was probed with anti-LMP1 antibody 1G6 followed by goat α-rat IgG HRP. The bands corresponding to LMP1 and the immunoglobulin heavy (H) and light (L) chains are indicated on the left. (B) Protein was extracted from B-cell lines 39.415 and 3959.48 with controls Raji and BJAB (the number of cells indicated below each track) using boiling mix and samples western blotted for LMP1 using IG6. The transgenic LMP1 (tgLMP1) is slightly smaller than Raji LMP1 (rLMP1) [3].

Carcinomas were induced in transgene positive and negative sibling controls (NSC) in the transgenic PyLMP1 line 53 (PyLMP1.53) [3], by topical treatment with chemical carcinogens [13]. These tumours could be readily established in culture; some retained a cuboidal, squamous morphology while others developed a spindle morphology with more transformed growth characteristics (additional files 1&2, supplementary table S1). LMP1 was difficult to extract from these epithelial cells (as with transgenic skin tissue [5]), suggesting an association with the cytoskeleton and necessitating the use of a urea extraction protocol. LMP1 expression was detected by immunoprecipitation and western blotting in several, but not all of the transgene positive carcinoma cell lines developed (figure 1A). Nevertheless, the cell lines in which expression could not be detected maintained the transgene (not shown). There was no apparent correlation between the carcinoma grade, cell line phenotype and LMP1 expression. For example, cell line 53.278a, derived from an aggressive spindle cell carcinoma and showing rapid spindle cell growth in culture (see supplementary information table 1) showed LMP1 expression as did the more cuboidal cell line 234a (with highest LMP1 expression) derived from a grade 3 carcinoma. However, with cuboidal cell line 53.226b (grade 1 carcinoma) and spindle cell line 53.191 (grade 3 carcinoma), little or no LMP1 expression could be detected.

**Table 1:** Clonagenicity of the epithelial cell lines with dnLMP1

cell line	transgene	colonies per μg of DNA		colony ratio
		
	status	pGFPdnLMP1	pGFP	pGFPdnLMP1:pGFP
53.217	**-**	164	170.4	1 : 1.0

53.234a	**+**	8.8	41.6	1 : 4.7

53.204	**+**	0	4.2	-

53.191	**+**	14	42	1 : 3.0

53.226b	**+**	1.6	6	1 : 3.8

53.278a	**+**	46	112	1 : 2.4

53.278b	**+**	1.2	13.2	1 : 11.0

**Figure 2:**
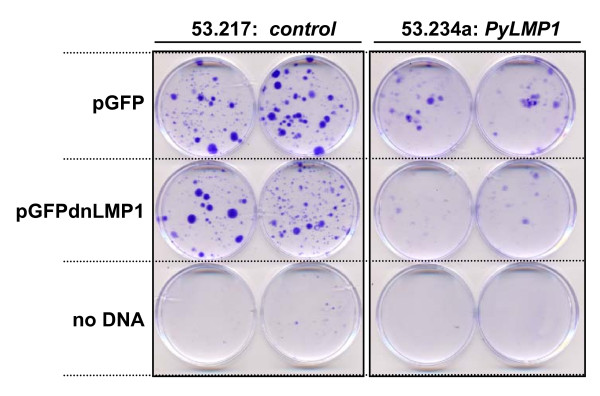
**GFPdnLMP1 inhibits the clonogenicity of PyLMP1 transgenic carcinoma cell lines**. Cell lines 53.217 (transgene negative control) and 53.234a (PyLMP1 transgenic) were transfected with 5 μg of either pGFP or GFPdnLMP1 or mock transfected (no DNA) in duplicate. After 24 hrs cells were passaged 1 to 8 and cultured for two weeks under G418 selection before fixing and staining with crystal violet. Colony numbers for these and further cell lines treated in the same way are given in Table 1.

The various cell lines (as shown) were transfected and grown for two weeks under G418 selection before fixing and staining with crystal violet (details in the legend to figure 2). The number of colonies per μg of DNA and the ratio of colonies for the GFPdnLMP1:GFP plasmids are tabulated.

Lymphomas arise spontaneously in aged mice of the transgenic line EμLMP1.39 (additional files 1&2, supplementary figure S1) in which LMP1 expression is directed to the lymphoid compartment [3]. Cell line 39.415 is a murine B-cell line developed from a lymphoma from transgenic line EμLMP1.39 (additional files 1&2, supplementary figure S2) showing readily detectable LMP1 expression (figure 1B). LMP1 expression in the 39.415 cell line is approximately 30-fold lower than the human BL cell line Raji (which harbours 50 to 60 copies of the EBV genome per cell). Cell line 3959.48 was established from a B-cell lymphoma arising in a bi-transgenic mouse harbouring EμLMP1 and EμEBNA-1 transgenes. It expresses readily detectable EBNA1 [14] and low levels of LMP1, with the latter at least 300-fold lower (on a per cell basis) than cell line 39.415 (figure 1B). Cell line 39.415 tends to grow in large clumps in culture, while 3959.48 grows as a single cell suspension or in small clumps, possibly reflecting LMP1 induced homotypic adhesion [15] and their relative levels of LMP1.

### Inhibition of LMP1 in the transgenic carcinoma cell lines

In order to inhibit LMP1 activity a dominant negative mutant of LMP1 which is defective in the LMP1 induced signalling pathways, termed LMP1^AAAG^, fused to GFP [16] denoted here as GFPdnLMP1 (or dnL as designation for transfected cells and clones) was introduced into the transgenic carcinoma cell lines. Using the parental GFP expression vector as control, six PyLMP1 transgenic carcinoma cell lines were transfected and one transgene negative control (figure 2, table 1). Following 2 weeks of plasmid selection, in all PyLMP1 cell lines the number of clones derived from pGFPdnLMP1 transfection was less than that from pGFP transfection, ranging from a 2.4 fold difference for (line 53.278a) to an 11 fold difference (line 53.278b) and in one cell line (53.204) no GFPdnLMP1 clones emerged. Furthermore, the pGFPdnLMP1 transfected clones tended to be smaller and less dense than the pGFP transfectants (figure 2). In contrast, clones of equivalent size and density were obtained in equal numbers for the two plasmids in the transgene-negative carcinoma cell line 53.217 (figure 2, table 1). This demonstrates that the pGFPdnLMP1 and pGFP plasmids were not toxic and of equal impact (if any) in an LMP1 negative carcinoma cell line. However, the data suggest that in all of the PyLMP1 transgenic cell lines, even those where LMP1 expression was low or undetectable (cell lines 53.278b, 53.226b, 53.191), dnLMP1 is inhibitory to clonagenicity. Clones derived in this manner were either cultured as a pool or individually isolated for further analysis from the transgene negative cell line 53.217 and two PyLMP1 positive cell lines 53.234a and 53.278a.

**Figure 3:**
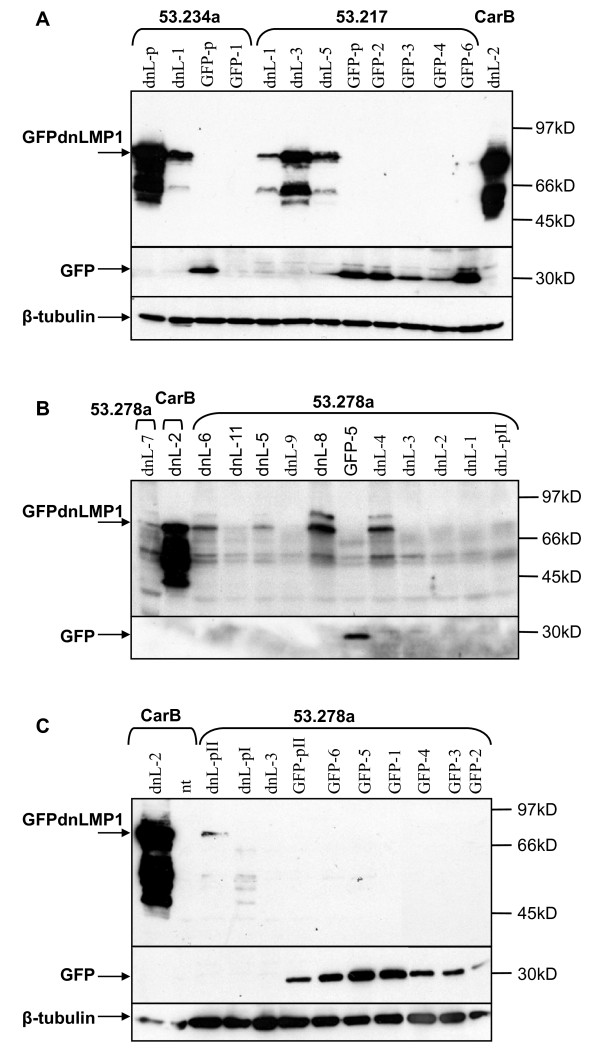
**Expression of GFP and GFPdnLMP1 in transfected carcinoma cells**. Transgene negative control cell lines 53.217 and CarbB, and transgenic PyLMP1 cell lines 53.234a and 53.278a, transfected with pGFP or pGFPdnLMP1 were sub-cloned to give clones denoted GFP and dnL (respectively). Protein samples from each cell line sub-clone (indicated by -number) or pool of clones (-p) were examined by western blotting, sequentially using anti-LMP1 (top of each panel), α-GFP (middle) and α-beta-tubulin (bottom), as indicated. (A) 100 μg of protein extract from sub-clones or pools of clones from the cell lines 53.217, CarB and 53.234a. (B) and (C) 50 μg protein extract from sub-clones or pools of clones (pI and PII) from the cell lines 53.278a and control CarB. Non-transfected parental cell line (nt).

Only one of six GFPdnLMP1 53.234a clones isolated could be established (referred to as 53.234dnL-1) while all six 53.217dnL clones were expanded. 10/12 clones of 53.278adnL were also established. This again reflects the inhibitory effect of dnLMP1 upon the clonagenicity of cell line 53.234a and to a lesser extent with cell line 53.278a. GFPdnLMP1 expression was confirmed in the single 53.234dnL-1 clone and in 3/3 tested 53.217dnL clones (figure 3A). For 53.278adnL clones, 5/10 showed clear GFPdnLMP1 expression (figure 3B and 3C and additional files 1&2, supplementary figure S3). GFP expression was confirmed in the majority of control pGFP transfected clones tested (figure 3).

**Figure 4:**
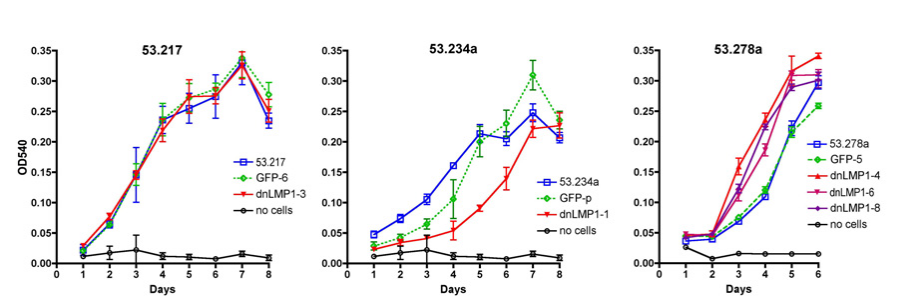
**Growth curves of GFP and GFPdnLMP1transfected carcinoma clones**. Clones of the transfected cell lines 53.217 (transgene negative; left graph), 53.234a (PyLMP1 transgene; middle graph) and 53.278a (Py LMP1 transgene; right graph) that showed the highest expression of GFP or GFPdnLMP1 were analysed for growth by neutral red assay and compared to the parental cell line in each case. Means of the four replicates (with SD) are plotted. Clone 53.234adnL-1 shows significantly different values from the parental cell line 53.234a (day 3: p < 0.0001, day 4: p < 0.0001, day 5: p < 0.0001, day 6: p = 0.0009). Similarly 53.278a clone values differ significantly from the parental 53.278a cell line (eg. for clone 53.278adnL-8: day 3: p = 0.0003, day 4: p < 0.0001, d5: p = 0.002).

The single 53.234dnL-1 clone established must have selectively overcome the inhibitory effect of dnLMP1 to some degree. In order to explore this further, clone 53.234dnL-1 was compared to clone 53.217dnL-3 (with highest expression of the 53.217 clones) for cell growth, against the parental cell lines and clones expressing only GFP. With the transgene negative cell line 53.217, clones expressing GFP or GFPdnLMP1 showed identical growth curves compared to the parental cell line (figure 4). However, the PyLMP1 positive clone 53.234dnL-1 showed significantly slower growth compared to both the parental cell line and GFP transfectants (figure 4). These data suggest that despite clone 53.234dnL-1 having been established under the selective pressure of dnLMP1 expression, i.e. inhibition of LMP1, the growth is nevertheless impaired compared to the parental cell line. Thus any genetic or epigenetic changes that have occurred in this cell clone to allow it to become established have not fully compensated for the blockade of LMP1 activity in cell growth.

We then examined the aggressive spindle cell line 53.278a which had shown least dependency upon LMP1 in the clonagenicity assay (table 1). Growth of three of the clones showing highest GFPdnLMP1 expression (53.278adnL-4, -6 and -8) were compared to the parental cell line and the highest GFP expressing control clone. The GFP clone 53.278aGFP-5 showed an identical growth rate to the parental cell line, while all three dnLMP1 clones revealed significantly accelerated growth rates (figure 4). These data demonstrate that enforced dnLMP1 expression in this cell line has selected for more rapidly growing clones presumably independent of LMP1 activity. The clone with highest GFPdnLMP1 expression, clone 53.278dnL-8 was assessed for tumourigenicity compared to the parental cell line, using syngeneic recipient mice. The clone retained the tumourigenic phenotype and in 3/4 subsequently derived tumours GFPdnLMP1 expression was maintained (additional files 1&2, supplementary figure S4).

### Inhibition of LMP1 in the transgenic B-cell lines

**Figure 5:**
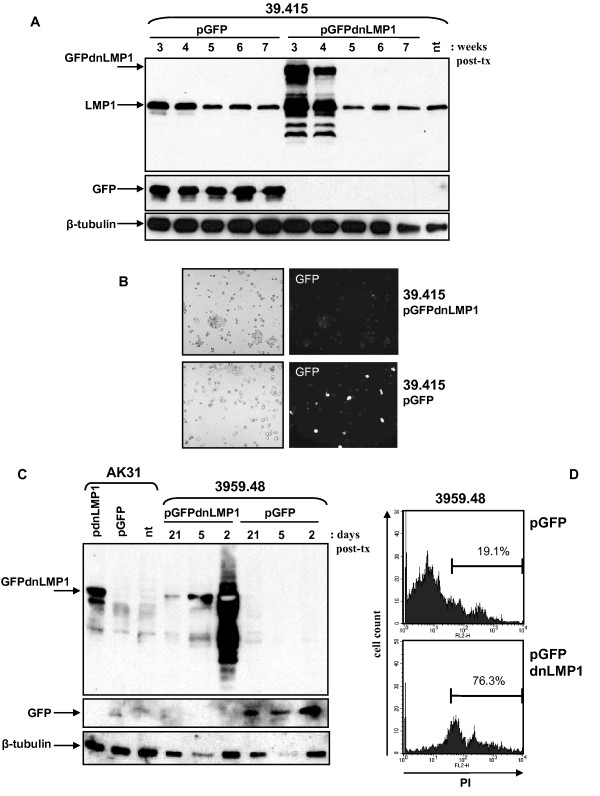
**GFPdnLMP1 expression is lost from EμLMP1 transgenic B-cell lymphoma cultures**. Transgenic EμLMP1 cell lines 39.415 and 3959.48, along with an EBV negative Akata cell line sub clone (AK31), transfected with pGFP or pGFPdnLMP1 were assayed for transfectant expression. (A) Protein extract from 5 × 10^5 ^39.415 transfected cells (and non-transfected control: nt) were examined by western blotting sequentially using anti-LMP1 (top panel), α-GFP (middle) and α-beta-tubulin (bottom), as indicated. Cell aliquots were collected after completion of selection at 3 weeks post transfection (post-tx) and then at weekly intervals (maintaining G418 selective pressure). (B) Bright field (left panel) and green fluorescence (right panel) visualized in pGFPdnLMP1 (top panel) or pGFP (bottom panel) transfected 39.415 cells at 3 weeks post transfection. (C) 40 μg of protein extract from 3959.48 and control AK31 transfected cells (and non-transfected control: nt) were examined by western blotting sequentially using anti-LMP1 (top panel), α-GFP (middle) and α-beta-tubulin (bottom), as indicated. Cell aliquots were collected at 2, 5 and 21 days post transfection (post-tx) for 3959.48 cells and at 12 weeks post-tx for AK31 cells (all under G418 selection). (D) At four weeks post pGFP or pGFPdnLMP1 transfection 3959.48 cells stained with propidium iodide (viable cells exclude staining, apoptotic cells stain) were analysed by flow cytometry, gating on GFP positive fluorescent cells only. Histograms show *y *axis (cell counts) and *x *axis (FL2-H, PI staining). The percentage of PI positive cells (of the GFP positive population) is indicated.

Inhibition of LMP1 activity in the tumour derived B-cell lymphoma cells lines 39.415 and 3959.48 was similarly assessed by transfection of the GFPdnLMP1 or GFP expression vectors. The antibiotic selection process was complete by 3 weeks post transfection at which point the cell lines were assayed for GFPdnLMP1 and GFP expression. Cells were harvested at weekly intervals for four weeks maintaining drug selection. With 39.415 cells, GFP expression could be detected in the control pGFP transfectants consistently for the four week period (figure 5A). However while clear GFPdnLMP1 expression was detected at 3 and 4 weeks post transfection, it disappeared from the transfected culture by 5 weeks post transfection. Similarly, clear green fluorescence could be seen in the pGFP transfectants (3 weeks post transfection) but only weak fluorescence in the pGFPdnLMP1 39.415 transfectants (figure 5B). In contrast, green fluorescence in both pGFP and pGFPdnLMP1 transfectants of the control EBV negative cell line AK31 was clearly visible (additional files 1&2, supplementary figure S5) and could consistently be detected by western to at least 12 weeks after transfection (figure 5C). With the 3959.48 cell line, similarly consistent GFP expression was seen in the controls, but GFPdnLMP1 expression could barely be detected in the transfected cultures at 3 weeks post transfection and was not detected by 4 weeks (not shown). Therefore earlier time points post transfection were examined. At two days post transfection of 3959.48 cells strong expression of GFPdnLMP1 was detected which was considerably reduced by 5 days post transfection and again only low level expression was detected by 3 weeks post transfection (despite plasmid selection), while control GFP expression in this cell line was constant (figure 5C).

Thus, either GFPdnLMP1 expression (unlike GFP alone) becomes repressed in the 39.415 and 3959.48 transfected cells or those cells expressing the dominant negative LMP1 protein are lost from the culture. In order to examine the viability of the GFPdnLMP1 expressing cells in the transfected, selected cultures, 3959.48 cells at four weeks post transfection were stained with propidium iodide (PI; apoptotic cells stain with PI while viable cells exclude the stain) and examined by flow cytometry. Of the pGFPdnLMP1 transfected cells 0.8% showed GFP fluorescence, of which 76.3% stained with PI (figure 5D). In contrast 6% of the pGFP transfected population showed GFP fluorescence of which 19.1% stained for PI. This suggests that the GFPdnLMP1 expressing cells were being eliminated from the population by apoptosis.

**Figure 6:**
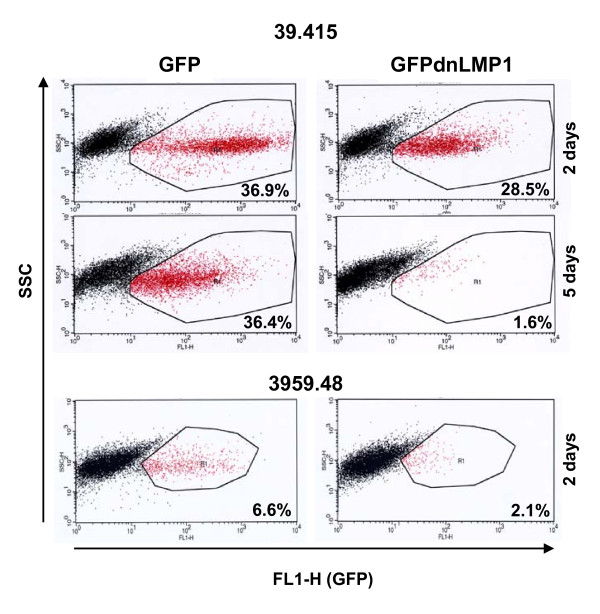
**GFPdnLMP1 expression is not compatible with the survival of EμLMP1 transgenic B-cell lymphoma lines**. Flow cytometric analysis of pGFP (left) or pGFPdnLMP1 (right) transfected 39.415 cells and 3959.48 cells as indicated. Cells were analysed two days and five days post-transfection (under G418 selection), dot plots showing *y *axis: side scatter (SSC) and *x *axis: FL1-H: green fluorescence (GFP). The gated population represents the GFP positive cells with the percentage indicated.

In order to look at earlier time points post transfection further, 39.415 and 3959.48 cells were examined by microscopy 24 hours after transfection. In these unselected cell populations bright fluorescent cells could clearly be seen in cultures transfected with both pGFP and pGFPdnLMP1, however there were fewer apparent in the latter and these often appeared morphologically unhealthy. In addition there was evidence of cells undergoing apoptosis in the pGFPdnLMP1 cultures (additional files 1&2, supplementary figure S6). GFP fluorescence in the transfected transgenic cells was also examined by flow cytometry. For cell line 39.415, the proportion of GFP expressing cells from 2 days post transfection to 5 days post transfection did not drop (36.9% to 36.4%). In contrast, the proportion of GFPdnLMP1 expressing cells dropped from 28.5% to 1.6% (figure 6). With 3959.48 cells 2 days post transfection, the proportion of GFP expressing cells was 6.6% compared to 2.1% for GFPdnLMP1 (figure 6).

These data demonstrate that both transgenic B-cell lines require the continued action of LMP1 for growth and survival, even in the cell line 3959.48 where LMP1 expression is very low.

## Discussion

In this study we have examined the consequences of inhibiting LMP1 activity in several cell lines which were derived from transgenic mice where LMP1 was the driving oncogene in the tumourigenic process. A dominant negative mutant of LMP1 which inhibits its signalling capacity was used with a view to future therapeutic drugs which might target LMP1 function in a competitive manner. We have explored the effects of inhibition in cells from established tumours, not upon cancer development, to reflect that in the clinical setting treatment is only initiated in patients with established tumours. Furthermore, in a number of these cell lines, LMP1 expression was low or undetectable and its continued function in the tumour cells was equivocal.

Inhibition of LMP1 activity in carcinoma cell lines established from LMP1 transgenic mice resulted in reduced clonagenicity (reflecting growth and survival of the cells at low density) in all of the cell lines tested. Interestingly, this was even the case in cell lines where LMP1 protein expression could not be detected, suggesting that there is a low-level (below detection) expression of LMP1 in these cell lines and that it still confers a growth advantage to the cells. This is relevant to NPC where LMP1 RNA can be detected in the majority of tumours but protein in only 30% to 50% of samples. This raises the possibility that the 50% to 70% of cases in which LMP1 protein is not detected may nevertheless express functioning LMP1.

Clonagenicity was not abolished in the carcinoma cell lines studied here (with the exception of one cell line). To varying degrees with the different lines, clones could be established and expression of dnLMP1 was shown for two LMP1 transgenic positive cell lines. Clone 53.234dnL-1 (derivative of 53.234a) must have undergone genetic or epigenetic changes to enable its establishment, but it nevertheless had slower growth characteristics than the parental cell line. As such, any changes it incurred did not fully compensate for LMP1 activity in the growth of the cell. By contrast 53.278adnL clones had more than compensated, evolving a faster growth pattern than the parental cell line. This cell line was derived from a spindle cell tumour, advanced and aggressive in nature and as such may have already undergone several changes *in vivo *to render LMP1 function redundant. One could therefore speculate that any LMP1 directed therapy with such a tumour (in the absence of other therapies) would force progression through selection, leading to drug resistant, aggressive escape mutants. Nevertheless, such a treatment could still potentially augment cytotoxic drug treatment.

With the cell lines and derived clones developed here we are now in a position to investigate the critical changes required in a tumour cell to allow it to overcome loss of LMP1 function. This knowledge could provide further targets to be used in combination with any LMP1 directed therapy.

The LMP1 transgenic B-cell lines examined could not tolerate inhibition of LMP1 activity, even in the 3959.48 cell line with very low LMP1 levels. Expression of dnLMP1 was either rapidly shut down in the transfected cells or those expressing cells were lost from the population. The kinetics would suggest the latter, as loss of dnLMP1 expression in the population could be seen after only a few population doublings, despite selection for plasmid retention. Moreover, after several weeks, the remaining GFPdnLMP1 positive cells showed evidence of undergoing apoptosis. With the loss of GFPdnLMP1 expression from the selected population, no expressing clones could be isolated. This is not to say that escape mutation is not possible in these B-cells, but perhaps occurs at too low a frequency to have been isolated in these assays.

Human EBV-associated tumours may have a more complex etiology than the transgenic tumours described here and indeed, several EBV-associated tumours show absolutely no expression of LMP1. However, in those EBV-associated tumours that do show LMP1 expression (if not consistently), like NPC and HD, it is reasonable to conclude from the wealth of data available on LMP1 activity, that it has been factorial in the development of the tumour, as it has in these transgenic mouse tumours. The data described here show that LMP1 continues to provide a growth advantage in established tumours even when expressed at very low level and provide proof of principle that in these tumour types LMP1 directed therapy could be effective.

## Conclusions

Generally, therapeutic drug resistance emerges following cancer treatments as a function of the number of tumour cells at the time of treatment as well as their potential ability to overcome the treatment. As a viral protein LMP1 diverts cellular processes to affect an altered growth programme of the cell and by secretion of induced factors, alter the cellular environment. It is not a cellular product and therefore not fundamentally essential to the cell. There are likely to be multiple mutational routes (of cellular genes) which could compensate for the loss of LMP1 function in the tumour cell and thus multiple possible routes for resistant cells to emerge following any LMP1 directed therapy. However, our results suggest that inhibition of LMP1 could be highly effective with some tumours and possibly stall others, even in EBV associated cancer types where detection of LMP1 is inconsistent, such as NPC. If combined with cytotoxic drugs, targeting LMP1 action could improve outcome in both epithelial and B-cell tumours.

## Methods

### EμLMP-1 and PyLMP1 transgenic lines and tumours

Transgenic mouse line EμLMP1 line 39 (EμLMP1.39), expressing low levels of LMP1 in the lymphoid compartment has been used in the studies described herein [3], maintained in the C57Bl/6 strain. Mice of this line develop lymphoma at an average age of approximately 20 months (supplementary information figure 1). Lymphoma incidence in the transgenic mouse line EμEBNA-1.59 expressing EBNA-1 in the lymphoid compartment, has been previously described [17]. Mice of line PyLMP1.53 (LMP1 of the EBV B95-8) express LMP1 in the epidermis and are maintained in the FVB mouse strain [3,13]. Carcinomas were induced in PyLMP1.53 mice using a standard single dose DMBA followed by 20 week TPA topical chemical carcinogen regime as previously described [13,18].

### Cell lines

Cell line 39.415 was developed following sequential *in vivo *passage of a B-cell tumour arising in mouse EμLMP1.39 number 415. After 3 passages in B6D2 strain immunocompetent mice, the tumour could be established in culture (supplementary information). Cell line 3959.48, expressing both LMP1 and EBNA-1 was established in culture following explant of a B-cell lymphoma from a bitransgenic mouse of the lines EμLMP1.39 and EμEBNA-1.59. B-cell lines were grown in RPMI supplemented with 10% FCS, 2 mM glutamine, 100 units/ml penicillin/streptomycin. Carcinoma cell lines were developed from primary carcinomas as described [13], grown in DMEM containing 10% FCS, 2 mM glutamine, 100 units/ml penicillin/streptomycin. CarB is a spindle-cell carcinoma cell line derived from a wild type mouse following DMBA/TPA chemical carcinogen treatment [19]. Raji is an EBV positive BL cell line, BL2B958 is an EBV negative BL cell line subsequently infected with EBV of the B95-8 strain, AK31 is an EBV negative derivative of the EBV positive Akata BL cell line.

### Protein extraction and western blotting

Protease inhibitors (complete mini, Roche), 1 mM phenylmethylsulfonyl fluoride and phosphatase inhibitors (10 μl/ml, phosphatase inhibitor cocktail II, Sigma) were freshly added to the protein extraction buffers. Proteins were extracted according to one of three protocols: [1] using urea protein extraction buffer (8M urea, 25 mM tris-HCl pH9.5, 5% (v/v) 2-mercaptoethanol), with incubation at 55°C overnight with agitation; [2] using RIPA buffer (150 mM NaCl, 50 mM tris-HCl pH7.5, 1% (v/v) triton, 1% (w/v) deoxycholic acid, 0.1% (w/v) SDS) followed by sonication; [3] alternatively counted cells were resuspended in PBS with protease inhibitors and sonicated and an equal volume of 2 × boiling mix was added (1 × boiling mix: 62.5 mM tris-HCl pH6.8, 2% (w/v) SDS, 5% (v/v) 2-mercaptoethanol, 10% (v/v) glycerol, trace bromophenol blue), heated to 95°C for 5 minutes for direct gel loading. Protein concentration was determined by Bradford assay (Biorad) or by 2D Quant assay (GE Healthcare). For SDS-PAGE, boiling mix was added to a 1× concentration to protein aliquots which were heated to 95°C for 5 minutes and loaded on to gels of 7.5%, 10% or 12.5%. Gels were blotted and blots were probed and washed as previously described [18]. Blots were incubated in 5% non-fat milk, 0.1% (v/v) Tween 20 in PBS with either 1:1000 anti β-tubulin (sc-9104, Santa Cruz), 1:100 1G6 (anti-LMP1) [20] or 1:500 anti-GFP (sc-8334, Santa Cruz) followed by 1:4000 of the appropriate IgG-HRP-conjugated secondary antibody (anti-rabbit sc-2030, anti-rat sc-2032 Santa-Cruz) and visualized by enhanced chemiluminescence (liteAblot kit, Euroclone).

### Immunoprecipitation

Equal quantities of urea-extracted protein samples were diluted at least 10-fold and made up to a total volume of 1 ml with NET-N pH8.0 (150 mM NaCl, 5 mM EDTA pH8, 50 mM tris-HCl pH8, 0.05% (v/v) NP-40) including protease and phosphatase inhibitors. To pre-clear, 70 μl of 50% (v/v) protein sepharose G (pre-washed) in NET-N buffer was added to each of the samples and rotated at 4°C for 2 hours. The samples were centrifuged at 10000 g for 10 mins at 4°C, and the pre-clear step was repeated with the supernatant using 30 μl of 50% (v/v) protein sepharose G. 4 μl of anti-LMP1 S12 [21] was added to the pre-cleared supernatant and rotated at 4°C overnight. 30 μl of 50% (v/v) protein sepharose G was added to each sample and rotated at 4°C for 30 mins. The samples were centrifuged at 10000 g for 10 mins at 4°C and the pellet was washed with 1 ml of NET-N pH8.0, followed by 1 ml of PBS with centrifugation at 10000 g for 1 min at 4°C. The antibody-antigen complexes were eluted from the beads with 30 μl of boiling mix at 95°C for 5 mins and centrifuged at 10000 g for 1 min prior to SDS-PAGE.

### Plasmids and transfection

The dominant-negative LMP1 plasmid pGFPdnLMP1 encoding an LMP1^AAAG ^mutant in which codons 204, 206, 208 and 384 have been changed from amino acids P, Q, T and Y to A, A, A and G and linked at the N-terminus to an in-frame enhanced-GFP tag, under the control of the CMV promoter, has been previously described [16]. It is abbreviated to dnL for cell subclones transfected with the plasmid. As control, pEGFP-C1 (Clontech) (abbreviated here to pGFP) encoding enhanced-GFP under the control of the CMV promoter has been used. B cells (typically 1 × 10^7^) were transfected with 10 μg of plasmid DNA by electroporation (960uF, 250 V), or "no DNA" as control, using a Biorad electroporater or an Amaxa nucleofector with solution V. One day after transfection cells were subjected to G418 selection (supplementary information table 1) and regarded as stably transfected (or post the selective process) when all no DNA controls cells were dead (2 to 3 weeks post transfection). Post selection cells were continually maintained in G418 thereafter. Epithelial cell lines were transfected in duplicate with either superfect (Qiagen) or metafectene lipid-based transfection reagents according to the manufacturer's instructions. Typically, one day after transfection cells were split 1:8 and then subjected to selection which was usually complete by 2 weeks (supplementary information table 1). Post selection clones were continually maintained in G418 thereafter.

### Clonagenicity assay with crystal violet

Cells were plated in 6 cm dishes, transfected with the appropriate plasmid and selected with G418. 14 days post-transfection, surviving colonies were stained with crystal violet solution (0.5% (w/v) crystal violet, 20% (v/v) ethanol in dH_2_O) at RT for 10 mins to 1 hour, washed gently with tap water and allowed to dry. The number of clones on each plate was counted directly.

### Cell growth assay with neutral red

Cells were seeded at a density of 500 cells per well (in quadruplicate sets for each daily count) in 96-well plates in 100 μl of medium. At daily intervals, cells were treated as follows: the medium was replaced in the wells to be analysed with pre-warmed neutral red-containing (40 μg/ml) medium and incubated at 37°C, 5% CO_2 _for 3 hours. The medium was removed, the cells were fixed with 100 μl of 1% (w/v) CaCl_2_, 0.5% (v/v) formaldehyde which was then removed and 100 μl of 1% (v/v) acetic acid 50% (v/v) ethanol was added to each well in order to liberate the dye from the viable cells that had incorporated stain. The plate was incubated at RT for 10-15 mins, rocked for 20-30 mins, then neutral red containing solutions were transferred to an empty plate and the absorbance was measured at 540 nm. Cells were assayed up to confluence (6 to 8 days). Statistical difference was calculated using a two sample T-test assuming equal variances.

### Flow cytometry

Cells were analysed using a FACScalibur flow cytometer (Becton Dickinson). Data was collected and analysed using CellQuest software (Becton Dickinson). Where possible, 10000 events (or GFP positive cells) were analysed. For GFP analysis cells were counted, washed twice with PBS and resuspended at 2 × 10^6 ^cells/ml in PBS prior to FACS analysis. Apoptosis was assessed by propidium iodide exclusion; cells were counted, washed twice with PBS and resuspended at 2 × 10^6 ^cells/ml in PBS. Typically 2 × 10^6 ^to 1 × 10^7 ^cells were used. Immediately prior to FACS analysis, 10 μl of 50 mg/ml propidium iodide (PI) solution was added per 1 ml of cell suspension.

## Competing interests

The authors declare that they have no competing interests.

## Authors' contributions

AH carried out the studies described, contributed to their design and helped to draft the manuscript. JBW conceived, coordinated and designed the study, performed the statistical analysis and drafted the manuscript. Both authors read and approved the final manuscript.

## Supplementary Material

Additional file 1 **This file contains the legends to the supplementary figures (S1 to S6) and supplementary table S1**. Click here for file

Additional file 2 **This file contains supplementary figures S1 to S6 and supplementary table S1**. Click here for file

## References

[B1] Crawford DH Biology and disease associations of Epstein-Barr virus Philos Trans R Soc Lond B Biol Sci 2001 356 461 473 10.1098/rstb.2000.0783 11313005 PMC1088438

[B2] Kieff E Rickinson AB Knipe DM, Howley PM, Griffin DEin Fields Virology 2001 Lippincott Williams & Wilkins, Philadelphia 2511 2573

[B3] Wilson JB Weinberg W Johnson R Yuspa S Levine AJ Expression of the BNLF-1 oncogene of Epstein-Barr virus in the skin of transgenic mice induces hyperplasia and aberrant expression of keratin 6 Cell 1990 61 1315 1327 10.1016/0092-8674(90)90695-B 1694724

[B4] Kulwichit W Edwards RH Davenport EM Baskar JF Godfrey V Raab-Traub N Expression of the Epstein-Barr virus latent membrane protein 1 induces B cell lymphoma in transgenic mice Proc Natl Acad Sci USA 1998 95 11963 11968 10.1073/pnas.95.20.11963 9751773 PMC21748

[B5] Stevenson D Charalambous C Wilson JB Epstein-Barr Virus Latent Membrane Protein 1 (CAO) Up-regulates VEGF and TGFa Concomitant with Hyperlasia, with Subsequent Up-regulation of p16 and MMP9 Cancer Res 2005 65 8826 8835 10.1158/0008-5472.CAN-05-0591 16204053

[B6] Young LS Murray PG Epstein-Barr virus and oncogenesis: from latent genes to tumours Oncogene 2003 22 5108 5121 10.1038/sj.onc.1206556 12910248

[B7] Miller WE Earp HS Raab-Traub N The Epstein-Barr virus latent membrane protein 1 induces expression of the epidermal growth factor receptor J Virol 1995 69 4390 4398 7769701 10.1128/jvi.69.7.4390-4398.1995 PMC189180

[B8] Charalambous CT Hannigan A Tsimbouri P McPhee GM Wilson JB Latent membrane protein 1-induced EGFR signalling is negatively regulated by TGF alpha prior to neoplasia Carcinogenesis 2007 28 1839 1848 10.1093/carcin/bgm055 17361012

[B9] Tsai J Lee JT Wang W Zhang J Cho H Mamo S Bremer R Gillette S Kong J Haass NK Discovery of a selective inhibitor of oncogenic B-Raf kinase with potent antimelanoma activity Proc Natl Acad Sci USA 2008 105 3041 3046 10.1073/pnas.0711741105 18287029 PMC2268581

[B10] Young LS Rickinson AB Epstein-Barr virus: 40 years on Nat Rev Cancer 2004 4 757 768 10.1038/nrc1452 15510157

[B11] Lo AK To KF Lo KW Lung RW Hui JW Liao G Hayward SD Modulation of LMP1 protein expression by EBV-encoded microRNAs Proc Natl Acad Sci USA 2007 104 16164 16169 10.1073/pnas.0702896104 17911266 PMC2042179

[B12] Mei YP Zhou JM Wang Y Huang H Deng R Feng GK Zeng YX Zhu XF Silencing of LMP1 induces cell cycle arrest and enhances chemosensitivity through inhibition of AKT signaling pathway in EBV-positive nasopharyngeal carcinoma cells Cell Cycle 2007 6 1379 1385 17507800 10.4161/cc.6.11.4274

[B13] Curran JA Laverty FS Campbell D Macdiarmid J Wilson JB Epstein-Barr virus encoded latent membrane protein-1 induces epithelial cell proliferation and sensitizes transgenic mice to chemical carcinogenesis Cancer Res 2001 61 6730 6738 11559544

[B14] Tsimbouri P Al-Sheikh Y Drotar ME Cushley W Wilson JB Epstein-Barr virus nuclear antigen-1 renders lymphocytes responsive to IL-2 but not IL-15 for survival J Gen Virol 2008 89 2821 2832 10.1099/vir.0.83296-0 18931080

[B15] Salcedo R Fuerstenberg SM Patarroyo M Winberg G The Epstein-Barr virus BNLF-1 membrane protein LMP1 induces homotypic adhesion mediated by CD11a/CD18 in a murine B-cell line, mimicking the action of phorbol ester J Virol 1991 65 5558 5563 1680199 10.1128/jvi.65.10.5558-5563.1991 PMC249062

[B16] Brennan P Floettmann JE Mehl A Jones M Rowe M Mechanism of action of a novel latent membrane protein-1 dominant negative J Biol Chem 2001 276 1195 1203 10.1074/jbc.M005461200 11031256

[B17] Wilson JB Bell JL Levine AJ Expression of Epstein-Barr virus nuclear antigen-1 induces B cell neoplasia in transgenic mice EMBO J 1996 15 3117 3126 8670812 PMC450254

[B18] Macdiarmid J Stevenson D Campbell DH Wilson JB The latent membrane protein 1 of Epstein-Barr virus and loss of the INK4a locus: paradoxes resolve to cooperation in carcinogenesis in vivo Carcinogenesis 2003 24 1209 1218 10.1093/carcin/bgg070 12807717

[B19] Diaz-Guerra M Haddow S Bauluz C Jorcano JL Cano A Balmain A Quintanilla M Expression of simple epithelial cytokeratins in mouse epidermal keratinocytes harboring Harvey ras gene alterations Cancer Res 1992 52 680 687 1370649

[B20] Nicholls J Hahn P Kremmer E Frohlich T Arnold GJ Sham J Kwong D Grasser FA Detection of wild type and deleted latent membrane protein 1 (LMP1) of Epstein-Barr virus in clinical biopsy material J Virol Methods 2004 116 79 88 10.1016/j.jviromet.2003.10.015 14715310

[B21] Mann KP Staunton D Thorley-Lawson DA Epstein-Barr virus-encoded protein found in plasma membranes of transformed cells J Virol 1985 55 710 720 2991591 10.1128/jvi.55.3.710-720.1985 PMC255052

