# Supplementary material for: Evaluation of LMP1 of Epstein-Barr virus as a therapeutic target by its inhibition
Source: Mol Cancer. 2010 Jul 9;9:184. doi: 10.1186/1476-4598-9-184 (PMC2913984; doi:10.1186/1476-4598-9-184)
Supplement: Additional file 1 — This file contains the legends to the supplementary figures (S1 to S6) and supplementary table S1. [file 1476-4598-9-184-S1.PDF]

Evaluation of LMP1 of Epstein-Barr virus as a therapeutic target by its inhibition

Adele Hannigan and Joanna B. Wilson

Additional file 1: supplementary information text, legends to supplementary table and figures.

Table S1 Cell lines

The squamous cell carinomas (SCC) were graded after histological examination into classical divisions from type 1: well differentiated through to type 4: poorly differentiated, with an increasing degree of invasion into subcutaneous tissue from type 1 to 4; and a final stage of spindle cell tumour (SpC) showing epithelial-mesenchymal transition (EMT) which is aggressively invasive and has lost markers of normal epithelium. The appearance of the epithelial cell lines soon after establishment in culture is indicated, with some cell lines becoming more spindle-like after multiple passages. The relative levels of LMP1 as detected by western blotting (shown in figure 1) are indicated. Column 6: epithelial cell lines were transfected with either superfect (S) or metafectene (M), and B-cells by electroporation (E), according to best efficiency for the cell line. The concentration of G418 used for selection is given. This was determined from a death curve for each cell line, using 50 to 100µg/ml increments and selecting the lowest concentration that achieved complete death of the culture within 10 days for epithelial cell lines and maximally 3 weeks for the B-cell lines.

Figure S1 Lymphoma incidence in EµLMP1 line 39

Fifteen founder transgenic mice were generated after injection of the EµLMP1 transgene sequences into B6D2.F2 zygotes from which eight viable mouse lines were generated as previously described {Wilson, 1990 #1}. EµLMP1 line 39 (EµLMP1.39), expressing very low levels of LMP1 in the lymphoid compartment has been used in the studies described herein. Lines with higher levels of expression showed poor survival and low fecundity, probably due to the broad pattern of LMP1 expression in these lines, not restricted to the lymphoid compartment {Wilson, 1990 #1}.

Despite low level expression of the LMP-1 encoding message in the normal thymus and spleen (before tumour development) of EµLMP1.39 mice {Wilson, 1990 #1}, the mice succumbed to lymphoma after a long latency period, in a similar fashion to that described by Kulwichit *et al.*{Kulwichit, 1998 #897}. Figure S1 shows a Kaplan-meier plot of lymphoma incidence, monitored in a cohort of EµLMP1.39 mice (n=39) compared to transgene negative controls (n=152). All mice were of C57Bl/6 strain. The curves are significantly different: log rank t test p = 0.0002. Lymphoma in the control mice showed a variety of presentations: spleen only, thymus only or multiple organ involvement, suggestive of neoplasias of different origins. Lymphoma in the EµLMP1.39 mice primarily presented in the mesenteric lymph nodes and/or spleen with variable involvement of the liver, peripheral lymph nodes and Peyers patches. Histopathological diagnosis of sections of these tissues revealed tumours of varying types with a predominance (approximately two thirds) of lymphoblastic lymphoma, with multi-organ involvement. Other cases were of histiocytic sarcoma (histiocytic

lymphoma) with one (out of 20 analysed) example of a mixed cell type lymphoma. Tumours were monoclonal carrying clear clonal rearrangements of the IgH locus. While most samples showed no T-cell receptor (TCR) rearrangements, one histiocytic lymphoma sample displayed both IgH rearrangements and a rearrangement of the TCRβ locus, suggesting that this tumour was likely of T-cell origin, while the majority are of B-cell origin. Confirmation of this was provided by flow cytometry showing that all tumours analysed (with the exception of the above mentioned histiocytic lymphoma) were B220 positive and CD3 negative (not shown).

Figure S2 Development of EµLMP1.39 lymphoma cell lines

The primary EµLMP1.39 tumours do not grow readily in culture, but can be established in culture following sequential *in vivo* passage. Tumours arising in EµLMP1.39 mice were transplanted into immuno-competent syngeneic (strain B6D2.F1) non-transgenic recipient mice. With the transfer of 10^6 primary tumour tissue cells, tumours would usually arise within 5 to 15 weeks in the recipients, presenting as clonal foci in the liver (top panel) and spleen (bottom panel) of the recipient. Serial *in vivo* passage of these tumours could be continued indefinitely, with time to tumour development in the recipients reducing to 2 weeks for certain tumours. On each passage, tumour cells from the foci were brought into culture and in this manner, cell lines were eventually established from several *in vivo* passaged tumours. Cell line 39.415 was established in culture following three passages *in vivo*. Cell line 3959.48 was derived from a tumour arising from a cross between EµLMP1.39 and EµEBNA-1 line 59 {Wilson, 1996 #158} and was established directly from the primary tumour (without *in vivo* passage).

Figure S3 Expression of GFPdnLMP1 in 53.278a dnLMP1 clone 8 (53.278dnLMP1-8)

Confocal microscope images of GFP fluorescence in the highest GFPdnLMP1 expressing clone of the 53.278a cell line, clone-8 (53.278adnL-8). GFP is shown in the left hand panel, the DAPI stained nuclei in the central panel and the merge of both images in the right hand panel.

Figure S4 GFPdnLMP1 expression in tumours derived from sub-cutaneous injections of 53.278a carcinoma cell lines

10^6 cells of the parental 53.278a cell line and the 53.278adnL-8 clone were injected subcutaneously into two mice and four mice (respectively). Three weeks post injection, tumours were apparent in all mice and these were collected and weighed (53.278a: mean weight = 192.8mg; 53.278adnL-8: mean weight = 271.6mg). Extracted protein samples from the tumours, along with cell lines samples of the parental cell line and clone 53.278adnL-8 (80µg protein) were separated by 10% SDS-PAGE, blotted and probed with an anti-LMP1 antibody. The GFPdnLMP1 band is indicated, along with the immunoglobulin heavy (H) and light (L) chain bands from the tumour tissue samples. Molecular weight markers are indicated. Three of the four tumours from clone 53.278adnL-8 have retained GFPdnLMP1 expression.

Figure S5 Localisation of GFP and GFPdnLMP1 in AK31 cells

AK31 (EBV negative) cells were transfected with either pGFP or pGFPdnLMP1. 24 hours post-transfection the cells were fixed and visualised with DAPI-containing mountant for analysis by fluorescence microscopy. The left hand panel of each row shows GFP fluorescence, the central panel shows DAPI stained nuclei and right hand panel shows the merge of the two images.

Figure S6 Apoptotic nuclei in 39.415 cells transfected with pGFPdnLMP1

39.415 cells were transfected with either pGFPdnLMP1 (A and B) or pGFP (C) (as indicated). 24 hours post-transfection the cells were fixed and stained with acridine orange. Apoptotic nuclei stained with acridine orange present with a characteristic beaded pattern as shown in (A) to higher magnification and indicated with an arrow in (B).
