# Supplementary material for: Evaluation of LMP1 of Epstein-Barr virus as a therapeutic target by its inhibition
Source: Mol Cancer. 2010 Jul 9;9:184. doi: 10.1186/1476-4598-9-184 (PMC2913984; doi:10.1186/1476-4598-9-184)
Supplement: Additional file 2 — This file contains supplementary figures S1 to S6 and supplementary table S1. [file 1476-4598-9-184-S2.PDF]

Evaluation of LMP1 of Epstein-Barr virus as a therapeutic target by its inhibition

Adele Hannigan and Joanna B. Wilson

Additional file 2: supplementary table (S1) and figures (S1 to S6)

Table S1 Cell lines

cell line	transgene	carcinoma grade	appearance	LMP1 expression	Trnsf. reagent	G418 µg/ml
53.217	negative	3	spindle, long pseudopodia	-	S	200
53.278b	PyLMP1	2	spindle, long pseudopodia	+/-	S	350
53.278a	PyLMP1	SpC	highly spindle like	+	S	350
53.226b	PyLMP1	1	cuboidal	-	M	350
53.226a	PyLMP1	2	mix of spindle and cuboidal cells	++		
53.191	PyLMP1	3	spindle, long pseudopodia	-	S	350
53.234a	PyLMP1	3	cuboidal	+++	S	350
53.204	PyLMP1	2	spindle, short pseudopodia	++	M	350
39.415	EµLMP1	B-cell lymphoma	cells cluster in large clumps	++++	E	200
3959.48	EµLMP1	B-cell lymphoma	rapid growth; cells cluster in clumps	+ (& EBNA-1 expression)	E	100

Figure S1 **Lymphoma incidence in EμLMP1 line 39**

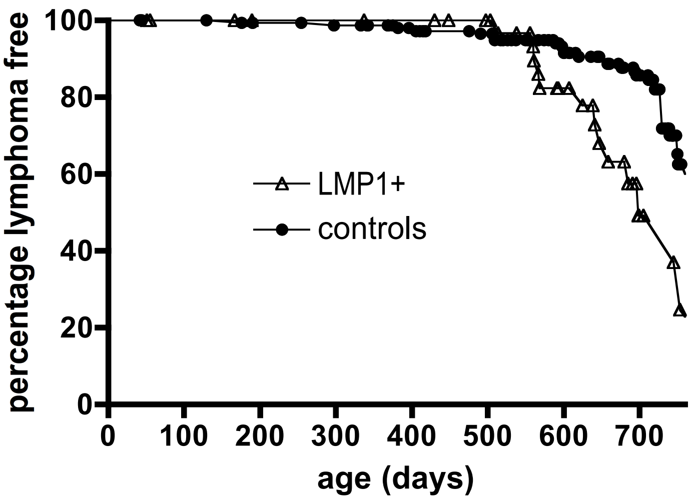

Figure S2 **EμLMP1.39 lymphoma transplantation**

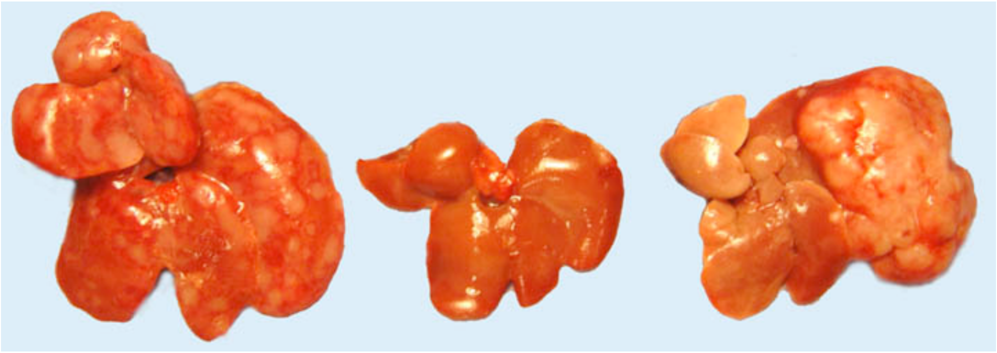

10^6 cells injected **CONTROL** **$3x10^3$ cells injected**

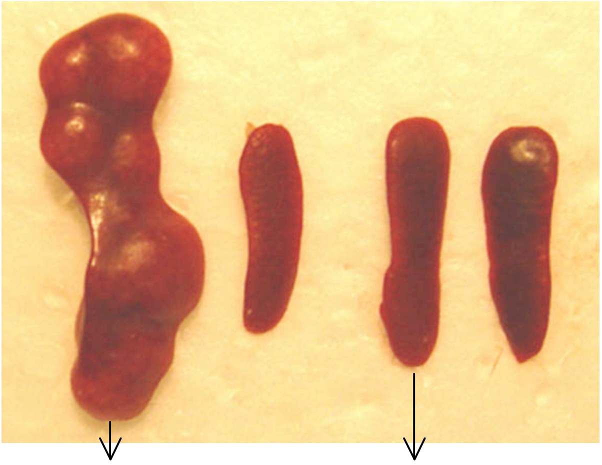

10^6 cells injected **$3x10^3$ cells injected**

CONTROL **CONTROL**

Figure S3 Expression of GFPdnLMP1 in 53.278a dnLMP1 clone 8 (53.278dnL-8)

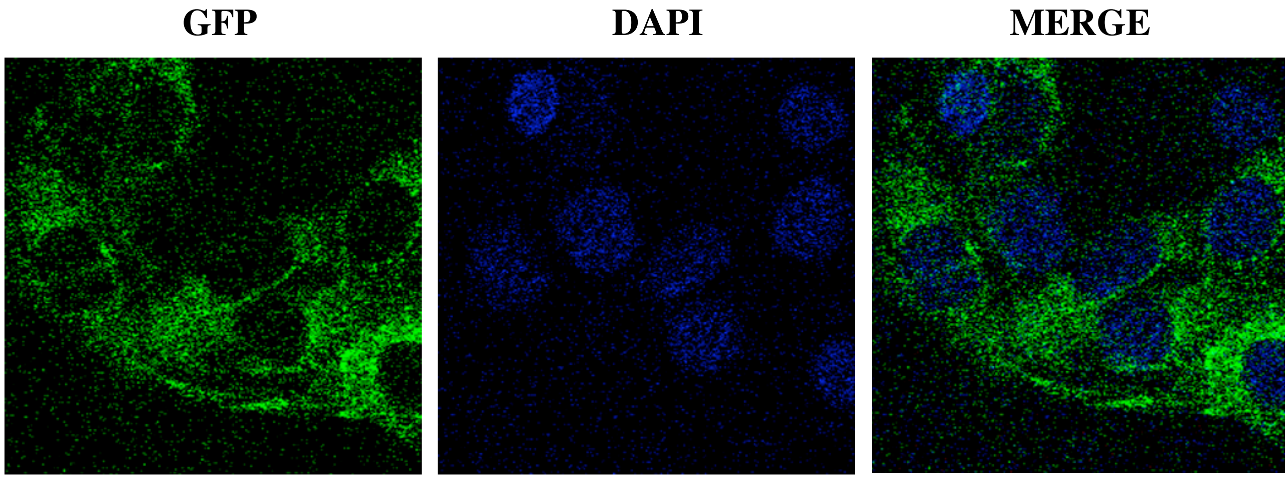

Figure S4 GFPdnLMP1 expression in tumours derived from sub-cutaneous injections of 53.278a carcinoma cell lines

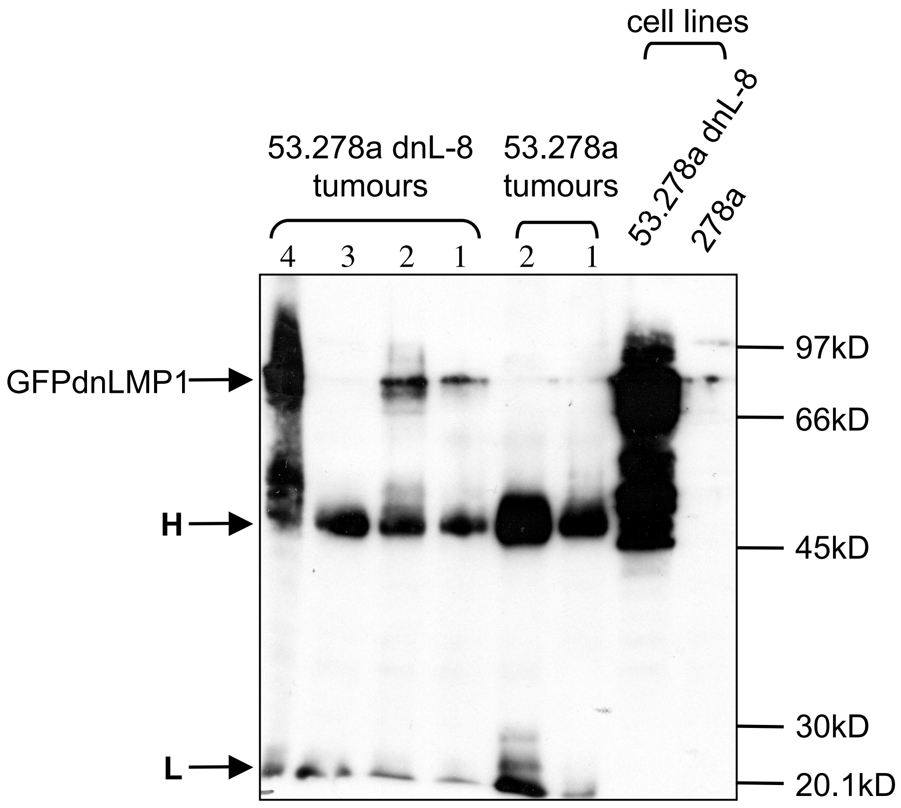

Figure S5 Localisation of GFP and GFPdnLMP1 in AK31 cells

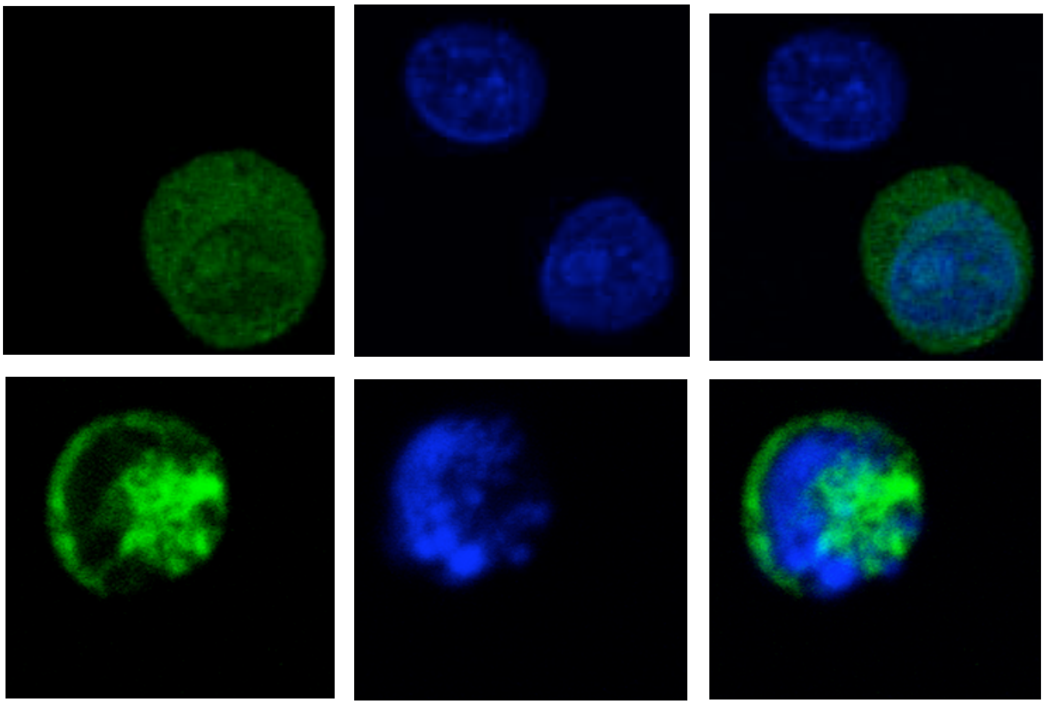

Figure S6 Apoptotic nuclei in 39.415 cells transfected with pGFPdnLMP1 stained with acridine orange

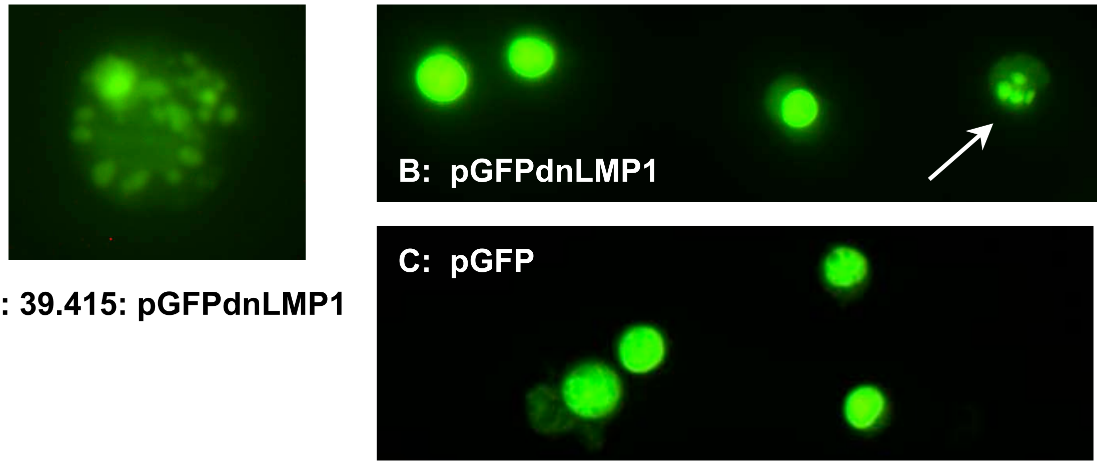

A: 39.415: pGFPdnLMP1
